# No Evidence of Mosquito Involvement in the Transmission of Equine Hepacivirus (Flaviviridae) in an Epidemiological Survey of Austrian Horses

**DOI:** 10.3390/v11111014

**Published:** 2019-11-01

**Authors:** Marcha Badenhorst, Phebe de Heus, Angelika Auer, Till Rümenapf, Birthe Tegtmeyer, Jolanta Kolodziejek, Norbert Nowotny, Eike Steinmann, Jessika-M.V. Cavalleri

**Affiliations:** 1 University Equine Clinic - Internal Medicine, Department for Companion Animals and Horses, University of Veterinary Medicine Vienna, Veterinärplatz 1, 1210 Vienna, Austria; marcha.badenhorst@vetmeduni.ac.at (M.B.);; 2 Institute of Virology, University of Veterinary Medicine Vienna, Veterinärplatz 1, 1210 Vienna, Austria; angelika.auer@vetmeduni.ac.at (A.A.); jolanta.kolodziejek@vetmeduni.ac.at (J.K.); norbert.nowotny@vetmeduni.ac.at (N.N.); 3 Institute for Experimental Virology, TWINCORE Centre for Experimental and Clinical Infection Research, Medical School Hannover (MHH) – Helmholtz Centre for Infection Research (HZI), Feodor-Lynen-Strasse 7, 30625 Hannover, Germany; birthe.tegtmeyer@twincore.de; 4 Department of Basic Medical Sciences, College of Medicine, Mohammed Bin Rashid University of Medicine and Health Sciences, Building 14, Dubai Healthcare City, Dubai, UAE; 5 Department of Molecular and Medical Virology, Ruhr-University Bochum, 44801 Bochum, Germany

**Keywords:** arbovirus, flavivirus, hematophagous arthropod, hepacivirus A, hepatitis, insects, mosquito-borne virus, virus transmission

## Abstract

Prevalence studies have demonstrated a global distribution of equine hepacivirus (EqHV), a member of the family Flaviviridae. However, apart from a single case of vertical transmission, natural routes of EqHV transmission remain elusive. Many known flaviviruses are horizontally transmitted between hematophagous arthropods and vertebrate hosts. This study represents the first investigation of potential EqHV transmission by mosquitoes. More than 5000 mosquitoes were collected across Austria and analyzed for EqHV ribonucleic acid (RNA) by reverse transcription quantitative polymerase chain reaction (RT-qPCR). Concurrently, 386 serum samples from horses in eastern Austria were analyzed for EqHV-specific antibodies by luciferase immunoprecipitation system (LIPS) and for EqHV RNA by RT-qPCR. Additionally, liver-specific biochemistry parameters were compared between EqHV RNA-positive horses and EqHV RNA-negative horses. Phylogenetic analysis was conducted in comparison to previously published sequences from various origins. No EqHV RNA was detected in mosquito pools. Serum samples yielded an EqHV antibody prevalence of 45.9% (177/386) and RNA prevalence of 4.15% (16/386). EqHV RNA-positive horses had significantly higher glutamate dehydrogenase (GLDH) levels (*p* = 0.013) than control horses. Phylogenetic analysis showed high similarity between nucleotide sequences of EqHV in Austrian horses and EqHV circulating in other regions. Despite frequently detected evidence of EqHV infection in Austrian horses, no viral RNA was found in mosquitoes. It is therefore unlikely that mosquitoes are vectors of this flavivirus.

## 1. Introduction

EqHV is one of 14 species belonging to the genus *Hepacivirus* in the family Flaviviridae [1]. This hepatotropic virus, also referred to as canine hepacivirus, non-primate hepacivirus and hepacivirus A, represents the closest related genetic homologue of hepatitis C virus (HCV) [1,2]. It is one of the novel viral agents, which has been associated with hepatitis in horses in recent years. EqHV infection typically results in subclinical hepatitis and transient, mild increases in liver-specific plasma biochemistry parameters [3].

Prevalence studies have demonstrated a global distribution of EqHV. The virus has been detected in horse populations across six continents, in countries including the USA, Brazil, South Africa, New Zealand, Korea, Japan, China, Scotland, France, as well as Austria’s neighboring countries Italy, Germany and Hungary [3,4,5,6,7,8,9,10,11,12,13,14,15,16]. However, apart from a single case of vertical transmission [17], natural routes of EqHV transmission remain elusive. Based on the frequent detection of EqHV RNA (prevalence up to 34.1%) [16], EqHV antibodies (prevalence up to 83.7%) [4] and the high EqHV prevalence in certain geographic regions and breeds [3,4,12,13], vertical transmission is unlikely to be the only route of natural infection. Phylogenetic clustering of EqHV isolates from individual horses within their respective herds also suggests a horizontal route of transmission [17]. Young horses subjected to intensive management practices appear to be particularly at risk [18,19]. HCV is known to spread by venereal transmission [20]. The spread of EqHV by the venereal route has been implicated in studies, which found the frequent occurrence of EqHV in a cohort of broodmares and breeding stallions [13] and a high frequency of EqHV RNA in horses bred for reproduction purposes [18]. The venereal transmission of EqHV remains speculative. However, comparable to HCV, experimental and iatrogenic transmission of EqHV by means of infected blood and blood products have been demonstrated [21,22,23].

Many known flaviviruses are horizontally transmitted between hematophagous arthropods and vertebrate hosts [24]. Examples include dengue virus, yellow fever virus (YFV), Japanese encephalitis virus (JEV), Zika virus (ZIKV), tick-borne encephalitis virus (TBEV), West Nile virus (WNV) and Usutu virus (USUV). Mosquito-borne viruses are transmitted by a vast range of mosquito species, depending primarily on the vector-competence of the mosquito species, the geographical region and susceptible vertebrate host species [24].

The primary aim of this study was to investigate whether various mosquito species, present in areas of EqHV endemicity in horses, carry EqHV nucleic acid and may transmit the virus horizontally between horses. Mosquitoes were collected across Austria and analyzed for EqHV RNA. Concurrently, the occurrence of EqHV was investigated—for the first time—in the horse population of Austria. The geographical locations of analyzed mosquito pools and study horses’ properties of origin were plotted on a map to determine proximity and compare EqHV statuses. Additionally, liver-specific plasma biochemistry parameters were compared between EqHV RNA-positive horses and EqHV RNA-negative control horses. Sequencing and phylogenetic analyses of Austrian EqHV strains were performed.

## 2. Materials and Methods

### 2.1. Study Design and Population

In this cross-sectional study, serum and plasma samples were collected for surveillance purposes from 386 horses in eastern Austria between July and October 2017. Sampled horses included patients of the University of Veterinary Medicine Vienna (Vetmeduni) Equine Clinic (*n* = 58), teaching horses of the Vetmeduni (*n* = 50) and privately owned, clinically unremarkable horses enrolled voluntarily (*n* = 278). The sample population consisted of various breeds and included 156 mares, 187 geldings, 42 stallions and one horse with the sex undisclosed. The horses’ ages ranged from 1 to 31 years (median age = 12.17 years). The geographic locations were recorded for the properties of origin of the horses. 

Considering an estimated population of 120 000 horses in Austria, the sample size was calculated. The expected prevalence of horses positive for EqHV RNA was set to 3.6%, which is the average prevalence of two surveillance studies in horse populations of various breeds and ages, performed in Germany (*n* = 433 horses; RNA prevalence = 2.5%) [3] and Italy (*n* = 1932 horses; RNA prevalence = 4.7 %) [6], respectively. Given a confidence interval of 95%, a sample size of 54 horses was required. The expected prevalence of EqHV antibody-positive horses was set to 31.4%—the antibody prevalence detected in the same German surveillance study [3]. Given a confidence interval of 95%, a sample size of 331 horses was required. Hence, the investigation of 386 horses was considered to be sufficient to estimate the prevalence of EqHV RNA and antibodies in Austrian horses. Sample size calculations were performed using Epitools Ausvet (epitools.ausvet.com.au/).

Data collection was approved by the Ethics Committee of the University of Veterinary Medicine Vienna, the Austrian Federal Ministry of Labor, Social Affairs, Health and Consumer Protection and the Austrian Federal Ministry of Education, Science and Research (study reference number BMWF- 68.205/0125-WF/V/3b/2017). 

### 2.2. Mosquito Collection

4039 mosquitoes collected in 2017 were pooled in 430 pools according to location, time of trapping and mosquito species. The mosquitoes were trapped across Austria and included both native and invasive species. In addition, 1004 mosquitoes were trapped in the 21^st^ Viennese district in August 2017, during a targeted mosquito and virus surveillance program following the identification of an USUV RNA-positive blood donor from that district [25]. These mosquitoes were pooled in 104 pools. The mosquito collection of the 21^st^ district was included in the study because a high number of investigated horses originated from this district, in which the Vetmeduni is located. A small number of mosquitoes from northeast Italy (*n* = 295; 32 pools; mainly invasive species), collected between June and October 2017, were also included in the analysis. In total, 5338 mosquitoes in 566 pools were investigated for the presence of EqHV nucleic acid. The following species were represented: *Aedes albopictus*, *Aedes japonicus*, *Aedes koreicus*, *Anopheles claviger, Anopheles maculipennis, Anopheles plumbeus, Culex hortensis, Culex pipiens, Culiseta annulata* and *Ochlerotatus geniculatus*.

### 2.3. Laboratory Analysis

#### 2.3.1. Detection of EqHV RNA in Mosquito Pools

Mosquito preparation was conducted as described previously [26]. From each mosquito pool homogenate, 140 µL was processed by automated nucleic acid extraction employing a QIAamp 12 Viral RNA Mini QIAcube Kit (Qiagen, Germany) according to the manufacturer’s instructions. Extracted RNA was transcribed into complementary deoxyribonucleic acid (cDNA) by applying the PrimeScriptRTMaster Mix Kit (TaKaRa, Kusatsu, Japan). All cDNA samples were stored at −20 °C before being used for RT-qPCR. For the SYBR Green based RT-qPCR, the SYBR^®^Premix Ex Taq™ II kit (TaKaRa, Kusatsu, Japan) was used, in combination with the previously described primers targeting the 5′ untranslated region (5′UTR) [5]. A standard curve for the quantification of RNA copies was assessed by serial dilution of a plasmid containing the EqHV 5′UTR based on the EqHV isolate NPHV-NZP-1 (JQ434001.1). Measurement of fluorescence was conducted with a LightCycler 480 (Roche, Mannheim, Germany). The limit of detection for this RT-qPCR assay was determined to be 7.86 viral copies/µL of homogenate. RT-qPCR analysis of mosquito pool homogenates was performed by the Department of Molecular and Medical Virology, Ruhr-University Bochum, Germany.

#### 2.3.2. Detection of EqHV RNA in Horse Serum

Viral RNA was extracted from 200 µL of each horse´s serum using the QIAamp 96 Virus QIAcube HT Kit (Qiagen, Germany) according to the manufacturer´s instructions. For screening, EqHV-specific nucleic acids were detected by RT-qPCR using primers and probes as described previously [5]. The RT-qPCR´s limit of quantification was established as 1.57 genome equivalents (GEs) per µL in the PCR reactions, which equates 7.85 × 10^2^ GEs per mL of horse serum. For absolute quantification, a serial dilution of a defined DNA plasmid standard was run in parallel with the positive samples. RT-qPCR analysis of horse serum samples was performed by the Institute of Virology, University of Veterinary Medicine Vienna, Austria.

#### 2.3.3. Detection of Anti-EqHV Non-Structural Protein 3 (NS3)-Specific Antibodies in Horse Serum

Serum samples were frozen between −20 °C and −80 °C prior to analysis for anti-EqHV non-structural protein 3 (NS3)-specific antibodies and shipped on dry ice to the laboratory in Germany. Samples were analyzed in duplicate for the presence of anti-EqHV NS3-specific antibodies, using the LIPS assay as described previously [27]. Relative light units (RLU) were measured with a plate luminometer (LB 960 XS3; Berthold, Bad Wildbad, Germany). The threshold value, above which samples were regarded as antibody-positive, was calculated for each plate by using the mean value plus three standard deviations (SDs) of an EqHV-negative horse serum sample. 

#### 2.3.4. Plasma Biochemistry

Plasma samples were frozen between −20 °C and −80 °C prior to analysis for GLDH, gamma-glutamyl transferase (GGT), bile acids and albumin concentrations at the Central Laboratory of the University of Veterinary Medicine Vienna. These parameters were measured in plasma samples of all EqHV RT-qPCR-positive horses, as well as in plasma samples of EqHV RT-qPCR-negative control horses (*n* = 45). The control horses were randomly selected from the group of privately owned, clinically unremarkable horses enrolled voluntarily. The laboratory’s reference ranges were used for data analysis.

### 2.4. Data Analysis

Plasma biochemistry results were assessed for normality using the Shapiro Wilk test. The independent samples *t*-test was used to compare normally distributed parameters. The Mann–Whitney U test was used to compare non-normally distributed parameters. A 95% confidence interval was set and *p* ≤ 0.05 was considered statistically significant. Statistical analysis was performed using IBM SPSS Statistics 24 and the applicable figure was generated using Graph Pad Prism 5. 

### 2.5. Sequencing and Phylogenetic Analyses

Sixteen EqHV RNA-positive samples were used for sequence analyses. Nested PCRs targeting parts of the 5′UTR, NS3 and NS5B domains were conducted as previously described [6,10] and sent for Sanger sequencing. Previously published complete genome sequences of EqHV and additional sequences of six isolates, which originated in France [12], were retrieved from the GenBank database. All sequences were aligned with MUSCLE and primer sequences were deleted. Phylogenetic analyses were conducted by using the maximum-likelihood method by MEGA7 [28] based on the general time reversible model [29]. Gamma distributed with invariant sites (G + I) was set for rates among sites with a number of six discrete gamma categories. Bootstrap replicates were set to 500. All sequences were uploaded to the NCBI database with accession numbers (MN475754 - MN475783).

## 3. Results

### 3.1. Detection of EqHV RNA in Mosquito Pools

**Figure 1:**
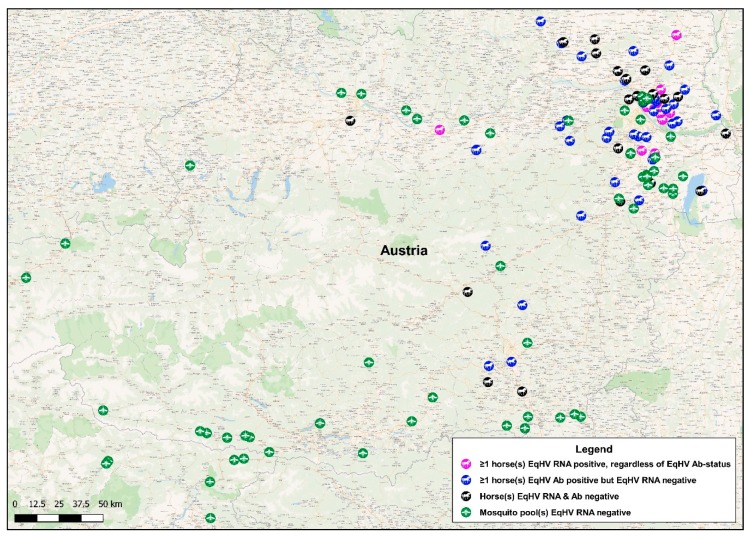
Geographical locations of sampling sites. A map of Austria indicating mosquito collection sites and the properties of origin of sampled horses. Green mosquito-icons each represents the collection site of a minimum of one mosquito pool. Magenta horse-icons each represents a property where at least one horse tested equine hepacivirus (EqHV) RNA-positive, regardless of the EqHV antibody-status of the horses on these properties. Blue horse-icons each represents a property where all horses tested EqHV RNA-negative, but at least one horse tested EqHV antibody-positive. Black horse-icons each represents a property where all horses tested EqHV RNA-negative, as well as EqHV antibody-negative.

No EqHV RNA was detected in any of the mosquito pools analyzed. The geographic locations of mosquito collection sites are indicated in Figure 1.

### 3.2. Detection of EqHV RNA in Horse Serum

EqHV RNA was detectable in 4.15% of serum samples (16/386). Viral loads in 11 of the samples ranged from 1 × 10^3^ to 9 × 10^6^ GEs per mL serum. Five horses showed RT-qPCR signals, which were below the assay’s level of quantification (<7.9 × 10^2^ GEs/mL serum). The 16 RT-qPCR-positive horses originated from 12 different properties, with between one and four RT-qPCR-positive horses per property (Table 1). The geographic locations of these properties are indicated in Figure 1.

### 3.3. Detection of Anti-EqHV NS3-Specific Antibodies in Horse Serum

The antibody prevalence in the study population was 45.9%, with 177 of the 386 samples antibody-positive. Based on the horses’ EqHV infection-state, the samples could be assigned to four categories: antibody-negative and RNA-negative (Abs–/RNA–); antibody-positive and RNA-negative (Abs+/RNA–); antibody-positive and RNA-positive (Abs+/RNA+) and antibody-negative and RNA-positive (Abs–/RNA+; Table 1). Two hundred and six samples (53.37%) tested negative for both EqHV antibodies and RNA. One hundred and sixty-four samples (42.49%) contained only antibodies, but no RNA. Thirteen samples (3.37%) contained both antibodies and RNA. Three samples (0.78%) contained only RNA, but no antibodies.

### 3.4. Plasma Biochemistry

EqHV RT-qPCR-positive horses (*n* = 16) had significantly higher plasma GLDH concentrations (*p* = 0.013) compared to EqHV RT-qPCR-negative control horses (*n* = 45; Table 2, Figure S1). All three of the EqHV RT-qPCR-positive horses which had GLDH levels above the laboratory reference range, were also EqHV antibody-positive (Abs+/RNA+). Two of these three horses were privately owned, clinically unremarkable horses enrolled voluntarily and one was admitted to the Vetmeduni Equine Clinic for follow-up examination, after a traumatic fracture of the fourth metatarsal bone. The horse had received tetanus antiserum two months before sample collection, following the acute trauma. Clinical findings did not give any indication for elevation of GLDH concentrations in this patient. All three of the horses which were acutely infected with EqHV (Abs–/RNA+), had GLDH levels well below the upper normal limit of the laboratory reference range. GGT, bile acids and albumin concentrations did not differ significantly between EqHV RT-qPCR-positive horses and EqHV RT-qPCR-negative control horses (*p* > 0.05; Table 2).

### 3.5. Sequence and Phylogenetic Analyses

For a molecular characterization of the EqHV positive samples, three nested PCRs were performed to target different conserved regions of the viral genome. Most likely as a result of variation in the viral load of samples, we were able to recover 12/16 partial 5′UTR sequences, 7/16 partial NS3 sequences and 11/16 partial NS5B sequences (Table 3).

**Figure 2:**
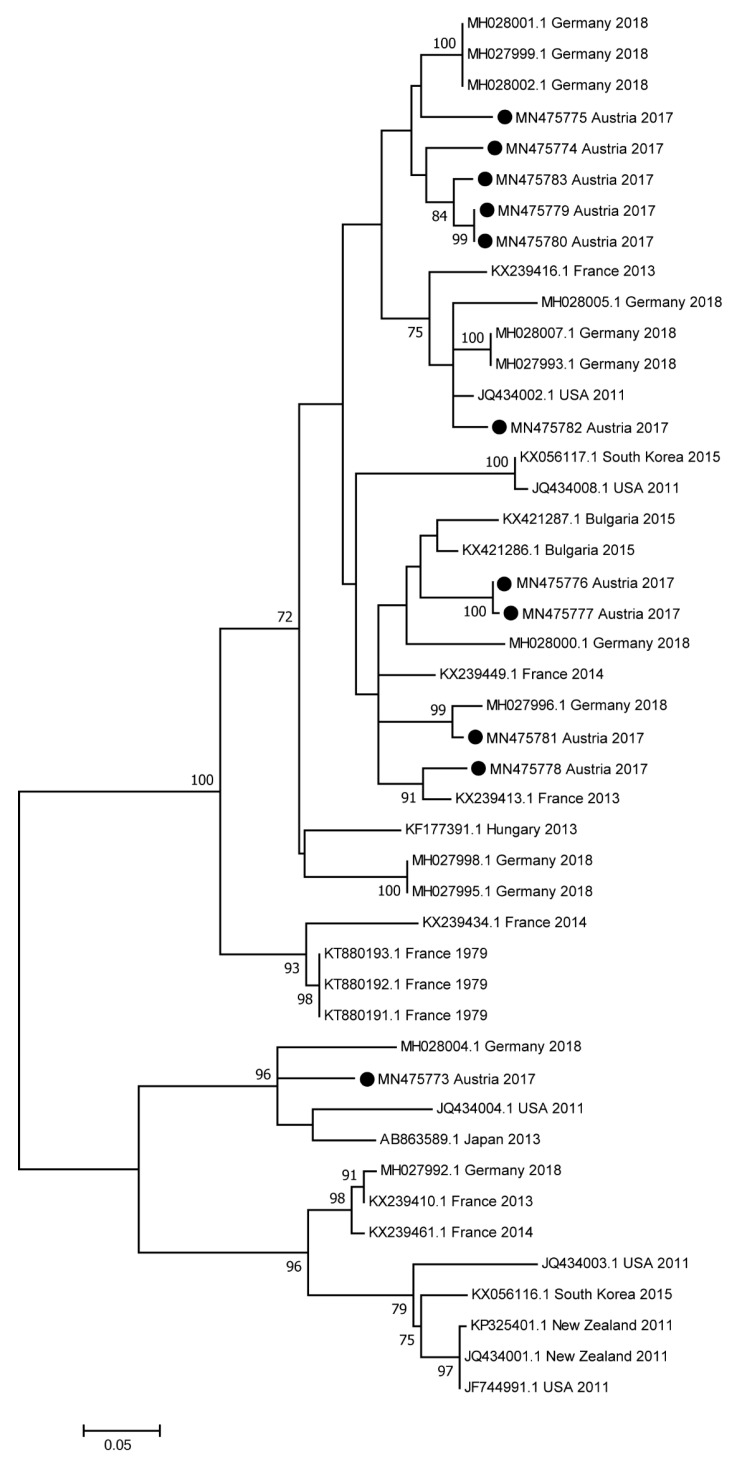
Maximum-likelihood phylogeny based on partial NS5B sequences of EqHV. In addition to sequences obtained from Austrian horses, the phylogenetic tree contains previously published complete genome sequences of EqHV retrieved from the GenBank database, as well as sequences of six isolates, which originated in France [12]. Different samples are identified with their accession number, country of origin and year of sampling or, if not applicable, year of publication. The analysis involved 45 nucleotide sequences. All positions containing gaps and missing data were eliminated, whereby a total of 258 positions were included in the final dataset. Bootstrap values <70% are not shown. The scale bar represents the number of substitutions per site. Black circles indicate samples obtained in this study.

Phylogenetic analysis based on partial NS5B sequences revealed only minor genetic distances between Austrian samples and previously published samples (Figure 2). Similarly, phylogenetic trees based on the 5′UTR (Figure S2) and on NS3 (Figure S3) visualize only minor differences between the sequences.

## 4. Discussion

The EqHV carrier-state of a large population of native and invasive mosquito species, collected across a large geographic area—the entire Austria and a small part of Italy—was investigated. This study also represents the first surveillance for EqHV in the horse population of Austria. The EqHV antibody prevalence (45.9%) and RNA prevalence (4.15%) detected in this population of Austrian horses were within previously reported ranges [4,16,30]. Despite the frequently detected evidence of EqHV infection in the equine study population, no indication of viral nucleic acid was found in mosquito populations throughout Austria and northeast Italy.

Mosquito surveillance programs were initiated in Austria in 2011, with the aim of detecting invasive mosquito species [31,32] and investigating the variety of viruses, which mosquitoes may carry and transmit to vertebrate hosts. Particularly the flaviviruses WNV and USUV have been detected [26,33,34,35,36,37]. For the current study, nucleic acid extracts from the entire 2017 mosquito-collection were investigated for the presence of EqHV RNA. The vast majority of mosquitoes were collected between June and October 2017, coinciding with the time of blood collection from the horses (July to October 2017). All mosquito species native to Austria were represented in the collection, as well as a large number of individuals from the invasive species *Aedes japonicus*. *A. japonicus* is rapidly dispersing in Austria [31], and was shown to be a flavivirus vector [35]. 

Mosquito-borne flaviviruses replicate within the invertebrate vector during the extrinsic incubation period—the time between uptake of an infectious blood meal by a female mosquito and subsequent viral transmission to the host [24]. A small percentage of infected female mosquitoes typically overwinter in frost-free areas, resulting in survival of the flavivirus [38]. Vertical transmission of flaviviruses is also frequently observed in infected female mosquitoes [26]. During the course of evolution, certain mosquito species developed “vector competence” for certain viruses. It was therefore essential to investigate a spectrum of mosquito species for the presence of EqHV RNA, since it is not known which mosquito species could be competent vectors for EqHV transmission. A large number of mosquito pools (*n* = 566), consisting of 5338 individuals from various species, representing five mosquito genera (*Aedes* spp., *Anopheles* spp., *Culex* spp., *Culiseta* spp. and *Ochlerotatus* spp.) contained no EqHV RNA. Based on these findings, mosquitoes are unlikely to play a role as vectors of EqHV. 

Comparable numbers of Austrian mosquitoes enabled us to determine the minimal infection rate (MIR) of WNV in previous investigations [36]. These results were in accordance with Czech and Hungarian studies, in which WNV-positive mosquitoes were detected in four of 650 pools [39] and in three of 645 pools [40], respectively. However, in areas with documented human or animal flavivirus infections, significantly higher numbers of mosquitoes were infested, as shown in [26] and [36] for WNV, and in [35] for USUV. We are therefore confident that the number of investigated mosquitoes was sufficient to also detect other flaviviruses.

Similar to HCV, transmission by means of infected blood and blood products has been demonstrated for EqHV [21,23]. Experimental infections of horses have been performed by intravenous administration of various volumes (range from 5 mL to 500 mL) of serum or plasma with varying viral loads (range from 3.92 × 10^3^ RNA copies/mL to 7.78 × 10^6^ RNA copies/mL) [21,23]. Considering this proven route of EqHV transmission, it remains to be determined whether EqHV can be mechanically transmitted by other hematophagous insects. The family Tabanidae (horse flies) is of particular importance as mechanical vectors of viruses such as equine infectious anemia (EIA) [41]. Painful bites, large lesions, persistent feeding behavior and the volume of blood left on their large mouthparts favors mechanical transmission [42]. The volume of blood retained on the mouthparts of a horse fly is approximately 5 to 25 nL [41,43]. Viral load in the blood of the host plays an important role in successful mechanical transmission of viruses. The minimum infectious dose of EqHV has not yet been determined. 

Natural and experimental EqHV infections typically result in transient elevation of concentrations of liver-specific enzymes, indicative of hepatic inflammation. Increased GLDH, sorbitol dehydrogenase (SDH), GGT and aspartate aminotransferase (AST) levels have been reported [3,21,23,44]. The increased plasma GLDH concentrations in EqHV RT-qPCR-positive horses are consistent with hepatocellular damage observed on liver histopathology of EqHV-infected horses [21]. Although plasma GLDH concentrations were significantly higher in EqHV RNA-positive horses compared to EqHV RNA-negative horses, the GLDH values of the majority of EqHV RNA-positive individuals were still within the laboratory reference range. Therefore, potential cases of EqHV should not only be monitored for liver-specific enzyme concentrations above references values, but also for deviations of enzyme concentrations from the individual horse’s baseline values. The timing of sampling is also of importance, with previous studies demonstrating that elevations in concentration of liver-specific enzymes typically do not occur during the acute phase of infection, but rather coincides with seroconversion [3,21]. Our findings are in accordance with this data.

In accordance with findings from other regions, the phylogenetic analyses revealed high similarity at nucleotide sequence-level of EqHV circulating in Austria, compared to other countries [4,6,10].

## 5. Conclusions

Despite evidence of EqHV infection being frequently detected in Austrian horses, no indication of viral nucleic acid was found in mosquito populations throughout the country. Consequently, mosquitoes are unlikely to play a role as vectors of this flavivirus. Nucleotide sequence differences between EqHV in Austria compared to EqHV of other origins are minor.

## Figures and Tables

**Table 1:** Information pertaining to the 12 properties where EqHV RNA-positive horses were identified, including the EqHV infection-state of the sampled populations.

Property		EqHV Infection-State							
		Abs-/RNA-		Abs+/RNA-		Abs+/RNA+		Abs-/RNA+	
Property ID	No. of Horses Sampled	*n*	%	*n*	%	*n*	%	*n*	%
1	12	4	33.33	7	58.33	1	8.33	0	0.00
2	10	4	40.00	5	50.00	1	10.00	0	0.00
3	20	13	65.00	6	30.00	1	5.00	0	0.00
4	21	7	33.33	10	47.62	3	14.29	1	4.76
5	14	2	14.29	11	78.57	1	7.14	0	0.00
6	32	16	50.00	14	43.75	1	3.13	1	3.13
7	3	1	33.33	1	33.33	1	33.33	0	0.00
8	20	13	65.00	6	30.00	1	5.00	0	0.00
9	13	7	53.85	5	38.46	1	7.69	0	0.00
10	50	34	68.00	15	30.00	0	0.00	1	2.00
11	1	0	0.00	0	0.00	1	100	0	0.00
12	1	0	0.00	0	0.00	1	100	0	0.00
All other	189	105	55.56	84	44.44	0	0.00	0	0.00
**Total**	**386**	**206**	**53.37**	**164**	**42.49**	**13**	**3.37**	**3**	**0.78**

**Table 2:** Specifications of the liver-specific biochemistry parameters glutamate dehydrogenase (GLDH), gamma-glutamyl transferase (GGT), bile acids and albumin measured in plasma samples of all EqHV RT-qPCR-positive horses (*n* = 16), as well as EqHV RT-qPCR-negative control horses (*n* = 45).

Parameter (Reference Range)	GLDH (<13 U/L)		GGT (<30 U/L)		Bile acids (<20 umol/L)		Albumin (2.4–4.5 g/dl)	
EqHV RT-qPCR Status	Positive	Negative	Positive	Negative	Positive	Negative	Positive	Negative
***n***	16	45	16	45	16	45	16	45
**Range (min–max)**	1.81–67.45	1.44–42.54	4–61	1–42	3–19	2–11	2.69–3.25	1.99–4.41
**Normal distribution**	No	No	No	No	No	No	Yes	Yes
**Median**	4.84	2.78	14.5	12	5	5	3.04	2.98
**Mean**	12.98	4.83	16.13	13.33	5.81	5.53	3.03	3.04
**Standard deviation**	20.27	6.83	13.27	7.92	3.94	2.16	0.16	0.52
**Parametric/Nonparametric test**	The Mann–Whitney U test		The Mann–Whitney U test		The Mann–Whitney U test		Independent samples *t*-test	
***p*-value**	*p* = 0.013 *		*p* = 0.434		*p* = 0.659		*p* = 0.855	

* EqHV RT-qPCR-positive horses had significantly higher plasma GLDH concentrations (*p* < 0.05) compared to EqHV RT-qPCR-negative control horses.

**Table 3:** The newly identified sequences which were submitted to NCBI and assigned with accession numbers. n.a.—not available.

Isolate	Accession Numbers		
	5‘UTR	NS3	NS5B
N40-17	MN475754	MN475766	MN475773
N87-17	MN475755	MN475767	MN475774
N107-17	MN475756	MN475768	MN475775
N147-17	MN475757	MN475769	MN475776
N154-17	MN475758	MN475770	MN475777
N201-17	MN475759	n.a.	MN475778
N234-17	MN475760	MN475771	MN475779
N235-17	MN475761	MN475772	MN475780
N265-17	MN475762	n.a.	MN475781
N351-17	MN475763	n.a.	MN475782
N364-17	MN475764	n.a.	MN475783
N403-17	MN475765	n.a.	n.a.

## Supplementary Materials

The following are available online at https://www.mdpi.com/1999-4915/11/11/1014/s1, Figure S1: Plasma GLDH concentrations of EqHV RT-qPCR-positive horses (n = 16) and EqHV RT-qPCR-negative control horses (n = 45), Figure S2: Maximum-likelihood phylogeny based on partial 5’UTR sequences of EqHV, Figure S3: Maximum-likelihood phylogeny based on partial NS3 sequences of EqHV.

## References

[1] Smith D.B., Becher P., Bukh J., Gould E.A., Meyers G., Monath T., Muerhoff A.S., Pletnev A., Rico-Hesse R., Stapleton J.T. (2016). Proposed update to the taxonomy of the genera *Hepacivirus* and *Pegivirus* within the Flaviviridae family. J. Gen. Virol..

[2] Pfaender S., Brown R.J., Pietschmann T., Steinmann E. (2014). Natural reservoirs for homologs of hepatitis C virus. Emerg. Microbes Infect..

[3] Pfaender S., Cavalleri J.M., Walter S., Doerrbecker J., Campana B., Brown R.J., Burbelo P.D., Postel A., Hahn K., Anggakusuma Riebesehl N. (2015). Clinical course of infection and viral tissue tropism of hepatitis C virus-like nonprimate hepaciviruses in horses. Hepatology.

[4] Badenhorst M., Tegtmeyer B., Todt D., Guthrie A., Feige K., Campe A., Steinmann E., Cavalleri J.M.V. (2018). First detection and frequent occurrence of Equine Hepacivirus in horses on the African continent. Vet. Microbiol..

[5] Burbelo P.D., Dubovi E.J., Simmonds P., Medina J.L., Henriquez J.A., Mishra N., Wagner J., Tokarz R., Cullen J.M., Iadarola M.J. (2012). Serology-enabled discovery of genetically diverse hepaciviruses in a new host. J. Virol..

[6] Elia G., Lanave G., Lorusso E., Parisi A., Cavaliere N., Patruno G., Terregino C., Decaro N., Martella V., Buonavoglia C. (2017). Identification and genetic characterization of equine hepaciviruses in Italy. Vet. Microbiol..

[7] Gemaque B.S., Junior Souza de Souza A., do Carmo Pereira Soares M., Malheiros A.P., Silva A.L., Alves M.M., Gomes-Gouvea M.S., Pinho J.R., Ferreira de Figueiredo H., Ribeiro D.B. (2014). Hepacivirus infection in domestic horses, Brazil, 2011–2013. Emerg. Infect. Dis..

[8] Kim H.-S., Moon H.-W., Sung H.W., Kwon H.M. (2017). First identification and phylogenetic analysis of equine hepacivirus in Korea. Infect. Genet. Evol..

[9] Lu G., Sun L., Xu T., He D., Wang Z., Ou S., Jia K., Yuan L., Li S. (2016). First Description of Hepacivirus and Pegivirus Infection in Domestic Horses in China: A Study in Guangdong Province, Heilongjiang Province and Hong Kong District. PLoS ONE.

[10] Lyons S., Kapoor A., Sharp C., Schneider B.S., Wolfe N.D., Culshaw G., Corcoran B., McGorum B.C., Simmonds P. (2012). Nonprimate hepaciviruses in domestic horses, United Kingdom. Emerg. Infect. Dis..

[11] Matsuu A., Hobo S., Ando K., Sanekata T., Sato F., Endo Y., Amaya T., Osaki T., Horie M., Masatani T. (2015). Genetic and serological surveillance for non-primate hepacivirus in horses in Japan. Vet. Microbiol..

[12] Pronost S., Hue E., Fortier C., Foursin M., Fortier G., Desbrosse F., Rey F.A., Pitel P.H., Richard E., Saunier B. (2017). Prevalence of Equine Hepacivirus Infections in France and Evidence for Two Viral Subtypes Circulating Worldwide. Transbound. Emerg. Dis..

[13] Reichert C., Campe A., Walter S., Pfaender S., Welsch K., Ruddat I., Sieme H., Feige K., Steinmann E., Cavalleri J.M.V. (2017). Frequent occurrence of nonprimate hepacivirus infections in Thoroughbred breeding horses—A cross-sectional study for the occurrence of infections and potential risk factors. Vet. Microbiol..

[14] Reuter G., Maza N., Pankovics P., Boros A. (2014). Non-primate hepacivirus infection with apparent hepatitis in a horse—Short communication. Acta. Vet. Hung..

[15] Schlottau K., Fereidouni S., Beer M., Hoffmann B. (2019). Molecular identification and characterization of nonprimate hepaciviruses in equines. Arch. Virol..

[16] Tanaka T., Kasai H., Yamashita A., Okuyama-Dobashi K., Yasumoto J., Maekawa S., Enomoto N., Okamoto T., Matsuura Y., Morimatsu M. (2014). Hallmarks of hepatitis C virus in equine hepacivirus. J. Virol..

[17] Gather T., Walter S., Todt D., Pfaender S., Brown R.J., Postel A., Becher P., Moritz A., Hansmann F., Baumgaertner W. (2016). Vertical transmission of hepatitis C virus-like non-primate hepacivirus in horses. J. Gen. Virol..

[18] Figueiredo A.S., Lampe E., de Albuquerque P.P.L.F., Chalhoub F.L.L., de Filippis A.M.B., Villar L.M., Cruz O.G., Pinto M.A., de Oliveira J.M. (2018). Epidemiological investigation and analysis of the NS5B gene and protein variability of non-primate hepacivirus in several horse cohorts in Rio de Janeiro state, Brazil. Infect. Genet. Evol..

[19] Figueiredo A.S., de Moraes M.V.D.S., Soares C.C., Chalhoub F.L.L., de Filippis A.M.B., Dos Santos D.R.L., de Almeida F.Q., Godoi T.L.O.S., de Souza A.M., Burdman T.R. (2019). First description of Theiler’s disease-associated virus infection and epidemiological investigation of equine pegivirus and equine hepacivirus coinfection in Brazil. Transbound. Emerg. Dis..

[20] Terrault N.A., Dodge J.L., Murphy E.L., Tavis J.E., Kiss A., Levin T.R., Gish R.G., Busch M.P., Reingold A.L., Alter M.J. (2013). Sexual transmission of hepatitis C virus among monogamous heterosexual couples. The HCV partners study. Hepatology.

[21] Pfaender S., Walter S., Grabski E., Todt D., Bruening J., Romero-Brey I., Gather T., Brown R.J.P., Hahn K., Puff C. (2017). Immune protection against reinfection with nonprimate hepacivirus. PNAS.

[22] Postel A., Cavalleri J.-M.V., Pfaender S., Walter S., Steinmann E., Fischer N., Feige K., Haas L., Becher P. (2016). Frequent presence of hepaci and pegiviruses in commercial equine serum pools. Vet. Microbiol..

[23] Ramsay J.D., Evanoff R., Wilkinson T.E., Divers T.J., Knowles D.P., Mealey R.H. (2015). Experimental transmission of equine hepacivirus in horses as a model for hepatitis C virus. Hepatology.

[24] Huang Y.-J.S., Higgs S., Horne K.M., Vanlandingham D.L. (2014). Flavivirus-mosquito interactions. Viruses.

[25] Bakonyi T., Jungbauer C., Aberle S.W., Kolodziejek J., Dimmel K., Stiasny K., Allerberger F., Nowotny N. (2017). Usutu virus infections among blood donors, Austria, July and August 2017—Raising awareness for diagnostic challenges. Euro. Surveill..

[26] Kolodziejek J., Seidel B., Jungbauer C., Dimmel K., Kolodziejek M., Rudolf I., Hubálek Z., Allerberger F., Nowotny N. (2015). West Nile virus positive blood donation and subsequent entomological investigation, Austria, 2014. PLoS ONE.

[27] Burbelo P.D., Ching K.H., Klimavicz C.M., Iadarola M.J. (2009). Antibody profiling by Luciferase Immunoprecipitation Systems (LIPS). J. Vis. Exp..

[28] Kumar S., Stecher G., Tamura K. (2016). MEGA7: Molecular Evolutionary Genetics Analysis Version 7.0 for Bigger Datasets. Mol. Biol. Evol..

[29] Nei M., Kumar S. (2000). Molecular Evolution and Phylogenetics.

[30] Lyons S., Kapoor A., Schneider B.S., Wolfe N.D., Culshaw G., Corcoran B., Durham A.E., Burden F., McGorum B.C., Simmonds P. (2014). Viraemic frequencies and seroprevalence of non-primate hepacivirus and equine pegiviruses in horses and other mammalian species. J. Gen. Virol..

[31] Seidel B., Nowotny N., Bakonyi T., Allerberger F., Schaffner F. (2016). Spread of *Aedes japonicus japonicus* (Theobald, 1901) in Austria, 2011-2015, and first records of the subspecies for Hungary, 2012, and the principality of Liechtenstein, 2015. Parasite Vectors.

[32] Seidel B., Montarsi F., Huemer H.P., Indra A., Capelli G., Allerberger F., Nowotny N. (2016). First record of the Asian bush mosquito, *Aedes japonicus japonicus*, in Italy: Invasion from an established Austrian population. Parasite Vectors.

[33] Bakonyi T., Ferenczi E., Erdélyi K., Kutasi O., Csörgő T., Seidel B., Weissenböck H., Brugger K., Bán E., Nowotny N. (2013). Explosive spread of a neuroinvasive lineage 2 West Nile virus in Central Europe, 2008/2009. Vet. Microbiol..

[34] Camp J.V., Bakonyi T., Soltész Z., Zechmeister T., Nowotny N. (2018). *Uranotaenia unguiculata* Edwards, 1913 are attracted to sound, feed on amphibians, and are infected with multiple viruses. Parasite Vectors.

[35] Camp J.V., Kolodziejek J., Nowotny N. (2019). Targeted surveillance reveals native and invasive mosquito species infected with Usutu virus. Parasite Vectors.

[36] Kolodziejek J., Jungbauer C., Aberle S.W., Allerberger F., Bagó Z., Camp J.V., Dimmel K., de Heus P., Kolodziejek M., Schiefer P. (2018). Integrated analysis of human-animal-vector surveillance: West Nile virus infections in Austria, 2015–2016. Emerg. Microbes Infect..

[37] Pachler K., Lebl K., Berer D., Rudolf I., Hubalek Z., Nowotny N. (2014). Putative new West Nile virus lineage in *Uranotaenia unguiculata* mosquitoes, Austria, 2013. Emerg. Infect. Dis..

[38] Rudolf I., Betášová L., Blažejová H., Venclíková K., Straková P., Šebesta O., Mendel J., Bakonyi T., Schaffner F., Nowotny N. (2017). West Nile virus in overwintering mosquitoes, central Europe. Parasite Vectors.

[39] Bakonyi T., Ivanics E., Erdélyi K., Ursu K., Ferenczi E., Weissenböck H., Nowotny N. (2006). Lineage 1 and 2 strains of encephalitic West Nile virus, central Europe. Emerg. Infect. Dis..

[40] Rudolf I., Bakonyi T., Šebesta O., Mendel J., Peško J., Betášová L., Blažejová H., Venclíková K., Straková P., Nowotny N. (2014). West Nile virus lineage 2 isolated from *Culex modestus* mosquitoes in the Czech Republic, 2013: Expansion of the European WNV endemic area to the North?. Euro. Surveill..

[41] Issel C.J., Foil L.D. (2015). Equine infectious anaemia and mechanical transmission. Man and the wee beasties. Rev. Sci. Tech. OIE.

[42] Carn V.M. (1996). The role of dipterous insects in the mechanical transmission of animal viruses. Brit. Vet. J..

[43] Scoles G.A., Miller J.A., Foil L.D. (2008). Comparison of the efficiency of biological transmission of *Anaplasma marginale* (Rickettsiales. Anaplasmataceae) by *Dermacentor andersoni* Stiles (Acari: Ixodidae) with mechanical transmission by the horse fly, *Tabanus fuscicostatus* Hine (Diptera: Muscidae). J. Med. Entomol..

[44] Gather T., Walter S., Pfaender S., Todt D., Feige K., Steinmann E., Cavalleri J.M.V. (2016). Acute and chronic infections with nonprimate hepacivirus in young horses. Vet. Res..

