# Supplementary material for: No Evidence of Mosquito Involvement in the Transmission of Equine Hepacivirus (Flaviviridae) in an Epidemiological Survey of Austrian Horses
Source: Viruses. 2019 Nov 1;11(11):1014. doi: 10.3390/v11111014 (PMC6893842; doi:10.3390/v11111014)
Supplement: Supplementary file 1 [file viruses-11-01014-s001.zip › Supplementary figures/viruses-616576-suppl..pdf]

Supplementary Figures:

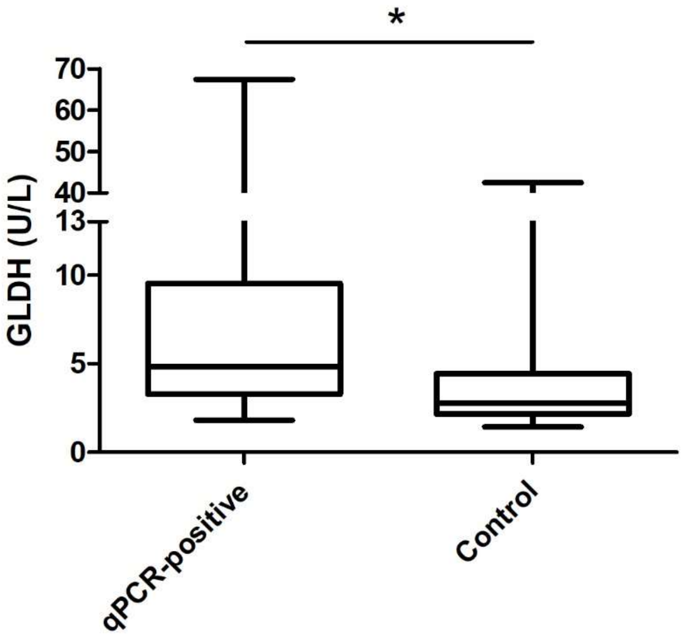

Supplementary Figure S1. Plasma GLDH concentrations of EqHV RT-qPCR-positive horses (n = 16) and EqHV RT-qPCR-negative control horses (n = 45). A significant difference in GLDH concentration of RT-qPCR-positive horses and control horses (P = 0.013) was determined by a Mann-Whitney U test, with P ≤ 0.05 considered statistically significant. The laboratory's normal reference range for plasma GLDH concentration was <13 U/L.

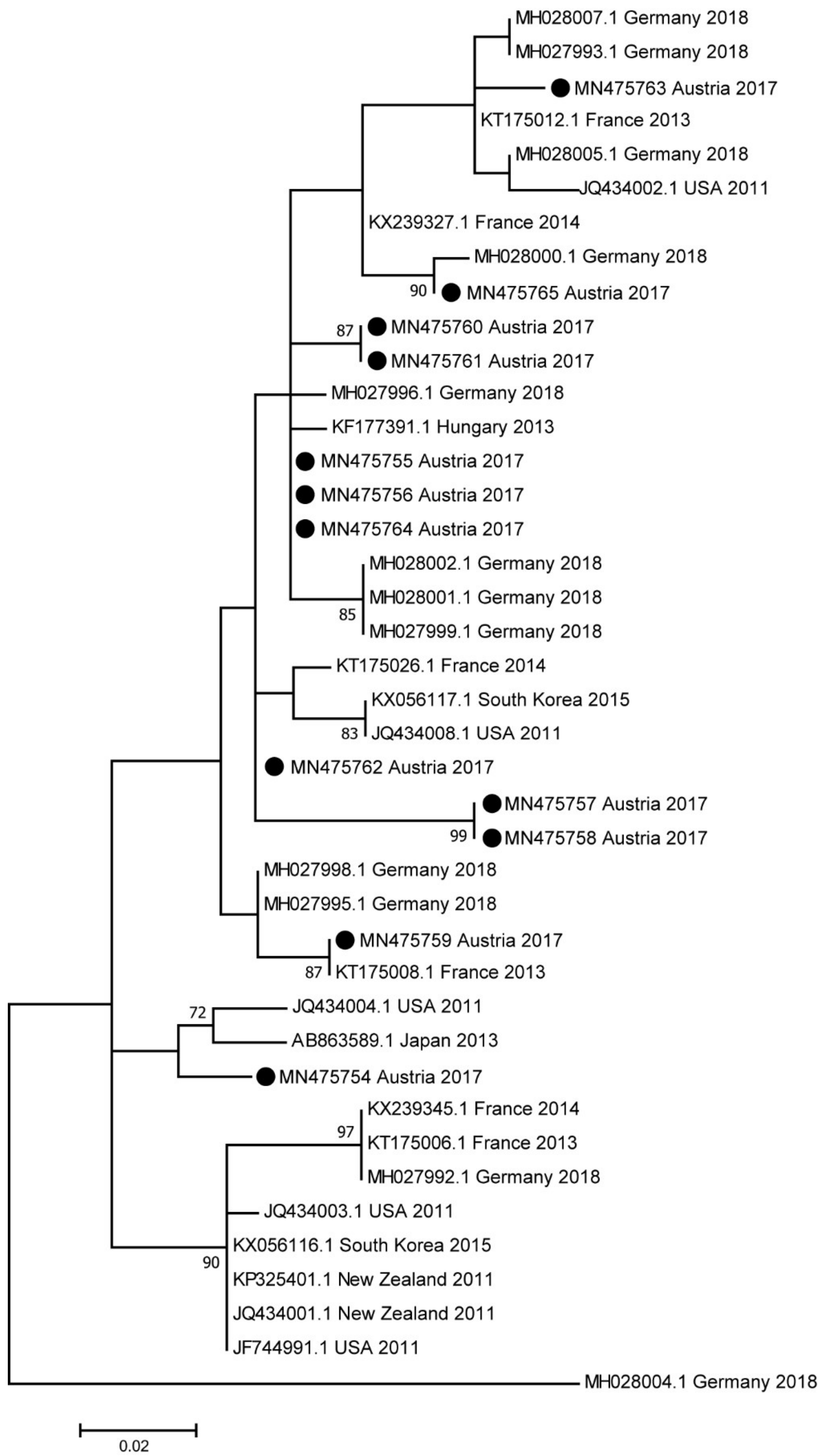

Supplementary Figure S2. Maximum-likelihood phylogeny is based on partial 5'UTR sequences of EqHV. In addition to sequences obtained from Austrian horses, the phylogenetic tree contains previously published complete genome sequences of EqHV retrieved from the GenBank database, as well as sequences of six isolates which originated in France [12]. The analysis involved 41 nucleotide sequences. All positions containing gaps and missing data were eliminated, whereby a total of 184 positions were included in the final dataset. Bootstrap values <70% are not shown. Different samples are identified with their accession number, country of origin and year of sampling or, if not applicable, year of publication. The scale bar represents the number of substitutions per site. Black circles indicate samples obtained in this study.

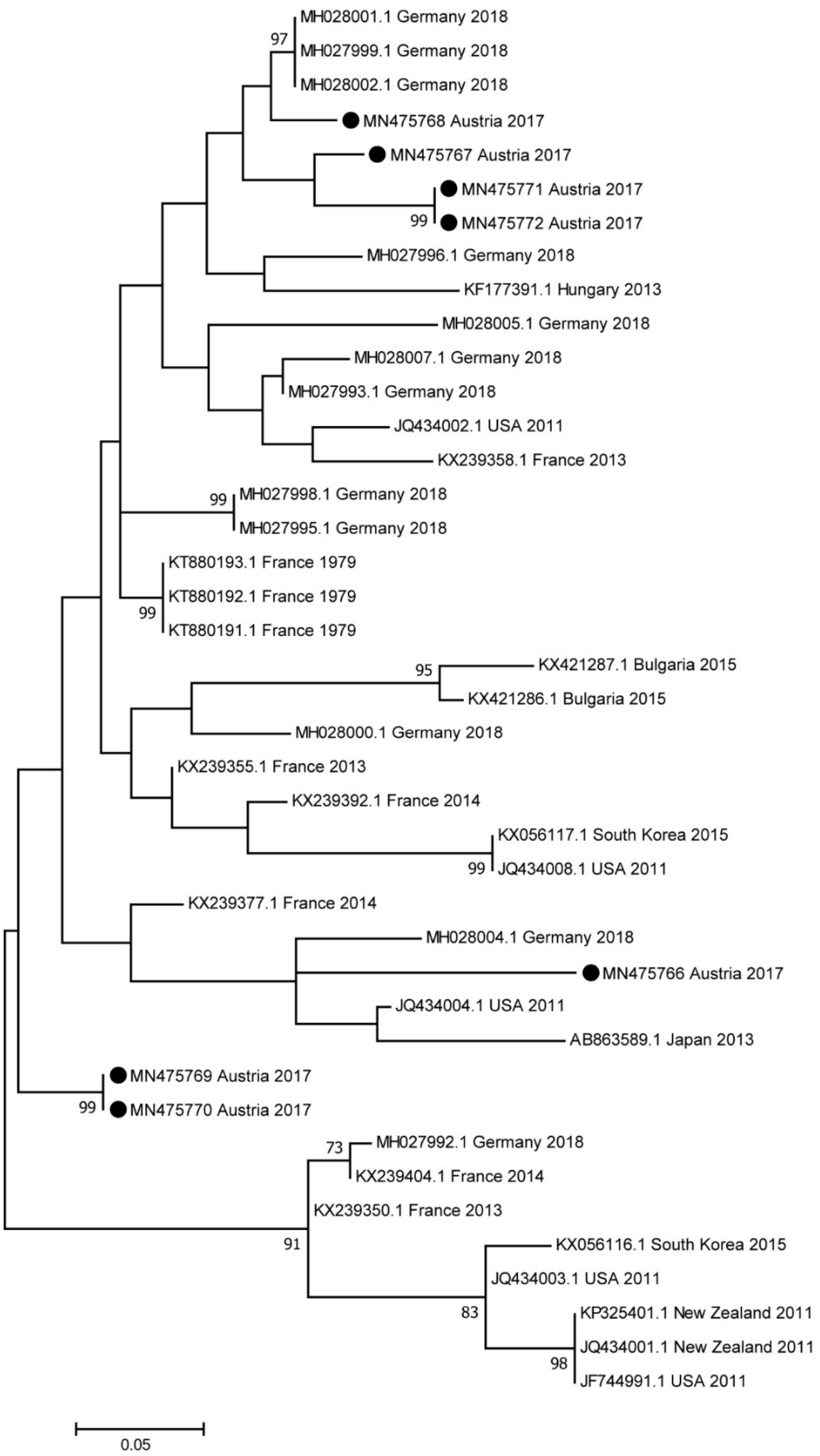

Supplementary Figure S3. Maximum-likelihood phylogeny is based on partial NS3 sequences of EqHV. In addition to sequences obtained from Austrian horses, the phylogenetic tree contains previously published complete genome sequences of EqHV retrieved from the GenBank database, as well as sequences of six isolates which originated in France [12]. The analysis involved 41 nucleotide sequences. All positions containing gaps and missing data were eliminated, whereby a total of 126 positions were included in the final dataset. Bootstrap values <70% are not shown. Different samples are identified with their accession number, country of origin and year of sampling or if not applicable year of publication. The scale bar represents the number of substitutions per site. Black circles indicate samples obtained in this study.
