# Supplementary figures and images for: No Evidence of Mosquito Involvement in the Transmission of Equine Hepacivirus (Flaviviridae) in an Epidemiological Survey of Austrian Horses
Source: Viruses. 2019 Nov 1;11(11):1014. doi: 10.3390/v11111014 (PMC6893842; doi:10.3390/v11111014)

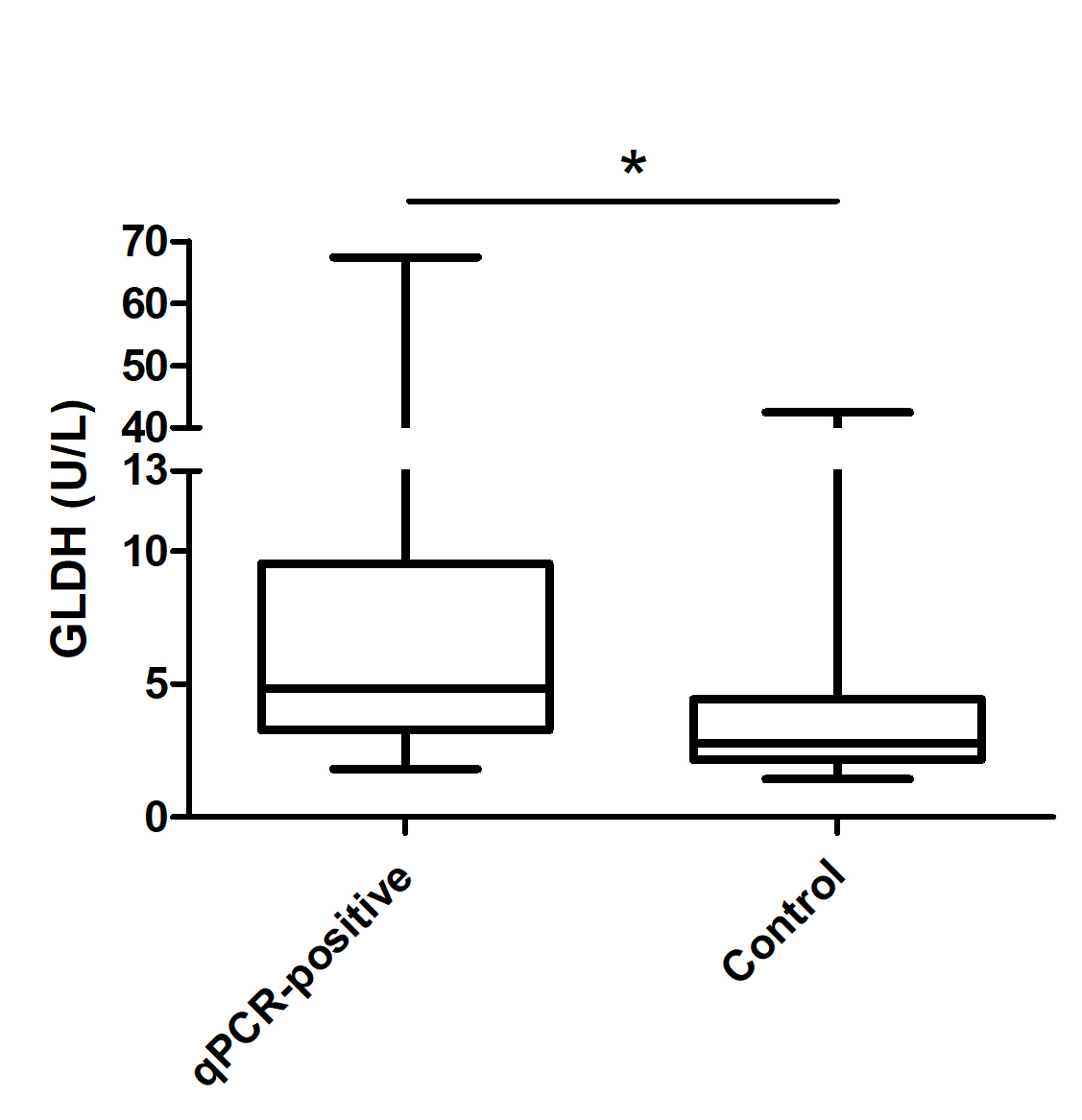

Supplement: Supplementary file 1 [file viruses-11-01014-s001.zip › Supplementary figures/21.10.19 Badenhorst et al 2019 Supp Fig S1.jpg]

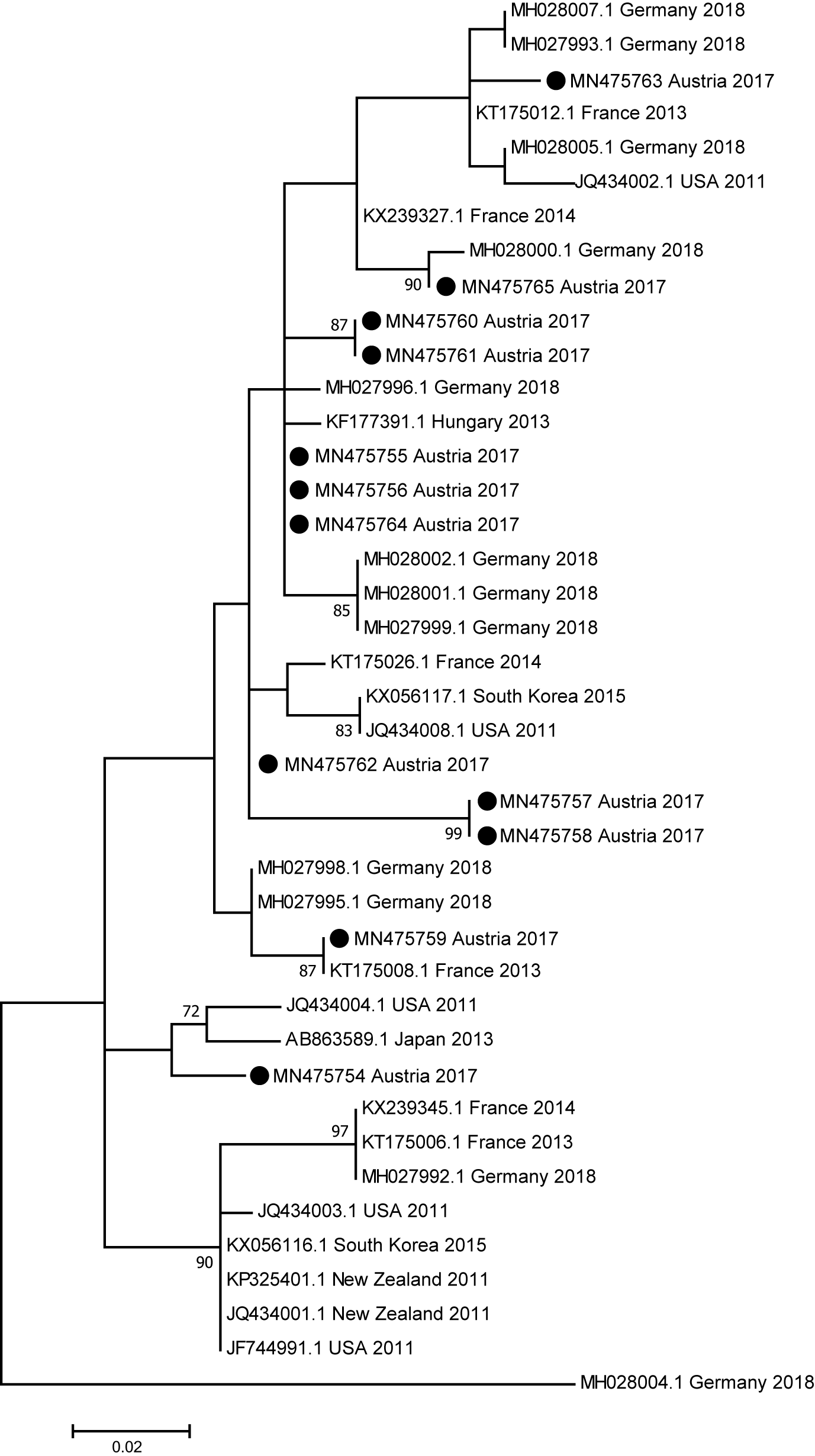

Supplement: Supplementary file 1 [file viruses-11-01014-s001.zip › Supplementary figures/21.10.19 Badenhorst et al 2019 Supp Fig S2.tif]

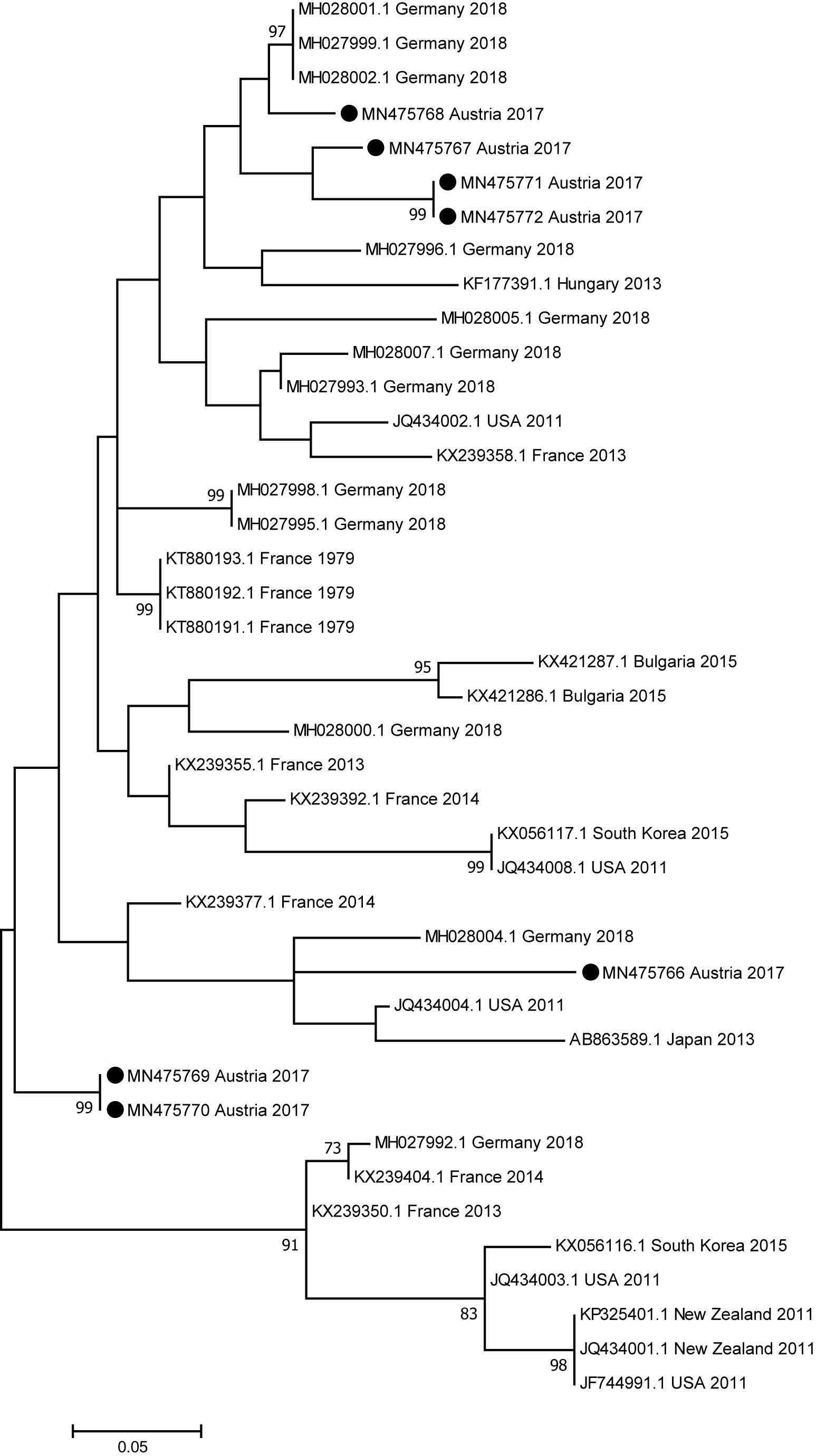

Supplement: Supplementary file 1 [file viruses-11-01014-s001.zip › Supplementary figures/21.10.19 Badenhorst et al 2019 Supp Fig S3.tif]
